# The Association Between Recurrence of Atrial Fibrillation and Revascularization in Patients With Coronary Artery Disease After Catheter Ablation

**DOI:** 10.3389/fcvm.2021.756552

**Published:** 2021-11-19

**Authors:** Xiaowei Chen, Jiangtao Zhao, Kui Zhu, Fen Qin, Hengdao Liu, Hailong Tao

**Affiliations:** Department of Cardiology, The First Affiliated Hospital of Zhengzhou University, Zhengzhou, China

**Keywords:** atrial fibrillation, radiofrequency catheter ablation, coronary artery disease, percutaneous coronary intervention, recurrence

## Abstract

**Aim:** The connection between revascularization for coronary artery disease (CAD) and the incidence of recurrent events of atrial fibrillation (AF) after ablation is unclear. This study aimed to explore the relationship between coronary revascularization and AF recurrence in patients who underwent radiofrequency catheter ablation (RFCA).

**Methods:** Four hundred and nineteen patients who underwent performed coronary angiography at the same time as RFCA were enrolled in this study. Obstructive CAD was defined as at least one coronary artery vessel stenosis of ≥75% and percutaneous coronary intervention (PCI) was recommended. Non-obstructive CAD was defined as coronary artery vessel stenosis of <75%. The endpoint was freedom from recurrence from AF after RFCA during the 24-month follow-up.

**Results:** In total, 102, 95, and 212 patients were undergone coronary angiography and diagnosed as having obstructive CAD, Non-obstructive CAD, and Non-CAD, respectively. During the 24-month follow-up period, patients without obstructive CAD were significantly more likely to achieve freedom from AF than patients with obstructive CAD (hazard ratio [HR]: 1.72; 95% confidence interval [CI]: 1.23–2.41; *P* = 0.001). The recurrence rate of AF was significantly lower in patients who underwent PCI than in those who did not (HR: 0.45; 95% CI: 0.25–0.80; *P* = 0.007). The multivariate regression analysis showed that the other predictors of AF recurrence for obstructive CAD were multivessel stenosis (HR: 1.92; 95% CI: 1.04–3.54; *P* = 0.036) and left atrial diameter (HR: 2.56; 95% CI: 1.31–5.00; *P* = 0.006).

**Conclusions:** This study suggests that obstructive CAD is associated with a higher rate of AF recurrence. Additionally, For patients with CAD, coronary revascularization is related to a lower recurrence rate of AF after RFCA.

## Introduction

Atrial fibrillation (AF) is the most prevalent sustained cardiac arrhythmia, and its incidence increases significantly with age (1). Patients with AF have a significantly higher risk of stroke and mortality (2). Coronary artery disease (CAD) is a common cardiovascular disease and a leading cause of mortality (3). Studies have revealed that the prevalence of CAD in patients with AF ranges from 17 to 46.5% (4–8). Multiple clinical risk factors, including hypertension, diabetes mellitus, increasing age, obesity, and sleep apnea, are shared by both diseases (9). Because the risks for both diseases overlap, CAD is likely to coexist with AF, and patients often undergo percutaneous coronary intervention (PCI) as treatment (10).

Radiofrequency catheter ablation (RFCA) is an important treatment strategy for patients with symptomatic drug-refractory AF (11). A previous meta-analysis proved that RFCA for AF had a higher efficacy rate than antiarrhythmic drug (AAD) therapy while having a lower rate of complications (12). However, the reported success rates of radiofrequency ablation in the treatment of AF are inconsistent. The success rate of atrial fibrillation after a single ablation procedure from different studies varies from 50 to 70% (13–16). Therefore, the success rate of RFCA is not very high. In addition to RFCA therapy, PCI is required for AF and CAD patients with severe coronary artery stenosis. Few studies have investigated the association between revascularization for CAD and recurrence of AF after RFCA. Our study aimed to investigate the relationship between CAD and AF recurrence in patients who underwent RFCA.

## Materials and Methods

### Study Population

**Figure 1:**
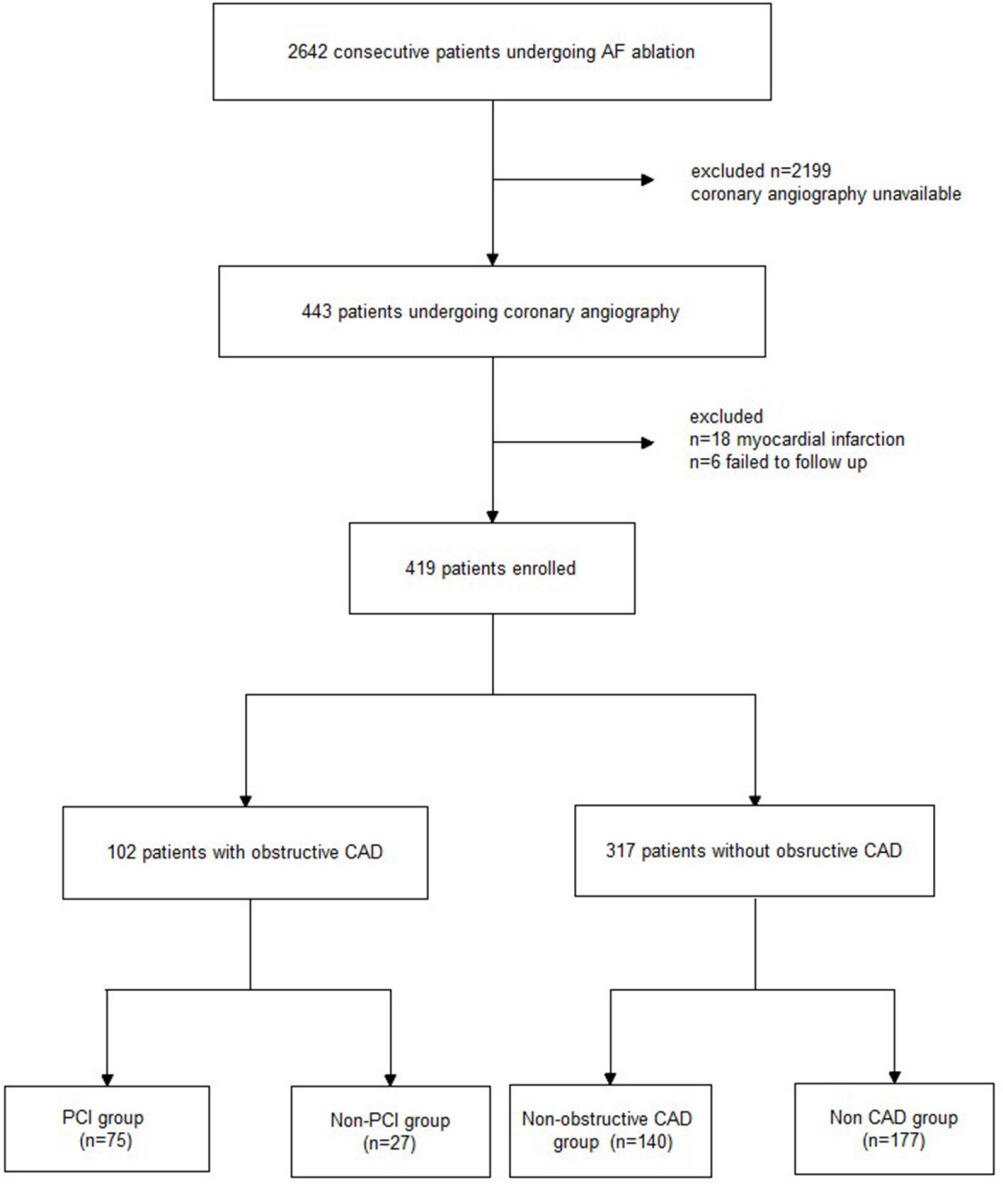
Study flowchart. AF, atrial fibrillation; CAD, coronary artery disease; PCI, percutaneous coronary intervention.

Between January 2017 and January 2019, 2,642 consecutive patients with AF had undergone RFCA in our center. Of these patients, 443 underwent coronary angiography 48 h before the RFCA because of suspected CAD. Of the 443 patients, 18 were excluded due to a previous history of myocardial infarction, and 6 patients could not be followed up. Finally, 419 patients were recruited for the analysis. Obstructive CAD and Non-obstructive CAD were confirmed by coronary angiography and PCI were recommended for patients with obstruction CAD. After RFCA, all patients were followed up and recurrence of AF was investigated in the next 24 months. Study design and flow are presented in Figure 1. This study was approved by the local ethics committee and complied with the Declaration of Helsinki. All patients provided informed consent for the ablation and PCI procedures, which were performed in accordance with the relevant guidelines.

### Ablation Procedure

For all enrolled patients, oral anticoagulant drugs (non-vitamin K antagonist oral anticoagulants, NOACs) were administered to the patients, and transoesophageal echocardiography was performed to rule out the possibility of an actual thrombus before the procedure. After the transseptal puncture, unfractionated heparin was administered at 100 U/kg and thereafter at 1,000 U/h to maintain the activated clotting time between 300 and 400 s. Under the guidance of the electroanatomical mapping system (CARTO, Biosense Webster, Diamond Bar, CA, USA), circumferential pulmonary vein isolation (PVI) was performed with the endpoint being the dissociation of all pulmonary vein (PV) potentials and a bidirectional conduction block from the atrium to the PV. Sinus rhythm was restored by cardioversion if AF was sustained after PVI. For patients with persistent AF, linear ablation with the endpoint being a bidirectional block and/or superior vena cava isolation was performed at the operator's discretion. An irrigated catheter was used in the procedure, and the radio frequency power output was usually up to 35–40 W with a maximum temperature of 45°C for each lesion and an irrigation flow of 20–25 mL/min. At the end of the procedure, the PVI and bidirectional block of the lines were evaluated, and the procedure was consolidated if necessary.

### Percutaneous Coronary Intervention

PCI was recommended when obstructive CAD (at least one coronary artery vessel had ≥75% stenosis) was identified by coronary angiography (intravascular ultrasound was required if visual critical stenosis was not confirmed). Non-obstructive CAD was confirmed if the coronary artery vessel had <75% stenosis. Before PCI, all patients received dual antiplatelet therapy, including aspirin (100 mg/d) combined with clopidogrel (75 mg/d). Anticoagulant drugs were not discontinued during the perioperative PCI period. Dual antiplatelet therapy combined with anticoagulant drug treatment was prescribed for 3 months. Then, a single antiplatelet drug (clopidogrel) combined with an anticoagulant drug was prescribed. Clopidogrel was continued for at least 12 months after PCI.

### Antiarrhythmic Drugs and Blanking Period Management

The first 3 months after RFCA is defined as the blanking period. Oral AADs were administered during the blanking period. Amiodarone was given orally at a dose of 600 mg/day for the first week, 400 mg/day for the second week, and then at a maintenance dose of 200 mg/day. Propafenone were prescribed if the patients could not tolerate amiodarone. Oral AAD treatment was used for 3 months after RFCA according to the physician's decision.

### Follow-Up and Complications

All patients were followed up at 1, 2, 3, and 6 months after the procedure every 3 months in the first year and every 6 months thereafter. At each hospital visit, participants underwent 24-h Holter monitoring and a 12-lead electrocardiogram (ECG). The participants were instructed to obtain an ECG or Holter ECG when they had symptoms indicative of arrhythmia recurrence. Any over-30s episode of atrial tachyarrhythmia (including AF/AT) on ECG was defined as AF recurrence. The study endpoint was freedom from any atrial tachyarrhythmia lasting >30 s after the ablation procedure. A total of four people had gastrointestinal bleeding during the follow-up. Two of them were in the revascularization group. One patient had melena and upper gastrointestinal bleeding in the first month and another patient had gastrointestinal bleeding in the second month after the RFCA. Clopidogrel + NOAC were prescribed instead of triple antithrombotic therapy. The other two patients were in the non-CAD group. After treatment, the patient's bleeding was controlled. No cerebral hemorrhage occurred during the follow-up period.

### Statistical Analysis

Continuous variables are presented as mean ± standard deviation. Categorical variables are presented as counts and percentages. Two-group comparisons of continuous variables were performed using the Student's *t*-test. Categorical variables in the two groups were compared using the χ^2^ test. Event-free survival in the two groups was estimated using a Kaplan-Meier analysis with the log-rank test. Arrhythmia recurrence and covariate associations were assessed using multivariate proportional hazard regression models. For each of the selected variables, hazard ratios with corresponding 95% confidence limits and Wald test *P*-values of the multivariable model were reported. In all analyses, a two-tailed *P*-value of 0.05 was regarded as statistically significant. All statistical analyses were performed using SPSS (version 17.0; IBM, Armonk, NY, USA).

## Results

### Patient Characteristics

Out of a total of 419 patients, obstructive CAD was diagnosed in 102 patients (24.3%) according to the extent of coronary stenosis. Patients with obstructive CAD had significantly higher rates of hypertension, dyslipidaemia, diabetes mellitus, and CHA2DS2-VASC scores than those without obstructive CAD. In addition, the obstructive CAD group had a greater left atrial diameter. The differences in the baseline characteristics are summarized in Table 1.

**Table 1:** Baseline characteristics of the patients with and without obstructive coronary artery disease.

	**All (*n* = 419)**	**With obstructive CAD (*n* = 102)**	**Without obstructive CAD (*n* = 317)**	***P-*value**
**Baseline characteristics**				
Age, mean ± SD, year	61 ± 10	63 ± 8	61 ± 11	0.215
Female sex, *n* (%)	288 (68.7)	65 (63.7)	223 (70.3)	0.210
BMI, mean ± SD, kg/m^2^	25.2 ± 2.8	25.6 ± 2.5	25.1 ± 2.9	0.171
Persistent AF, *n* (%)	187 (44.6)	42 (40.2)	146 (46.1)	0.389
Hypertension, *n* (%)	223 (53.2)	69 (67.6)	154 (48.6)	0.001
Dyslipidemia, *n* (%)	151 (36.0)	65 (63.7)	86 (27.1)	<0.001
Diabetes mellitus, *n* (%)	57 (12.9)	28 (22.5)	31 (9.8)	<0.001
Renal insufficiency, *n* (%)	5 (1.2)	2 (1.9)	3 (0.9)	0.599
CHA2DS2-VASC, mean ± SD	1.5 ± 1.2	1.9 ± 1.4	1.4 ± 1.2	0.001
Left atrial diameter, mean ± SD, mm	42.7 ± 3.6	43.4 ± 3.3	42.5 ± 3.7	0.029
LVEF, mean ± SD, %	63 ± 3	62 ± 3	63 ± 2	0.129
BNP, mean ± SD, pg/ml	78 ± 44	81 ± 49	77 ± 43	0.425
**Medication**				
β-blockers, *n* (%)	291 (69.5)	75 (73.5)	216 (68.1)	0.304
ACE/ARB inhibitor, *n* (%)	177 (42.2)	59 (57.8)	118 (37.1)	<0.001
Statins, *n* (%)	267 (63.7)	95 (93.1)	172 (54.3)	<0.001

*Values are reported as means ± SD or n (%). BMI, body mass index; AF, atrial fibrillation; LVEF, left ventricular ejection fraction; ACE, angiotensin converting enzyme; ARB, angiotensin receptor blocker; CAD, coronary artery disease; SD, standard deviation*.

### Association Between AF Recurrence and CAD

**Figure 2:**
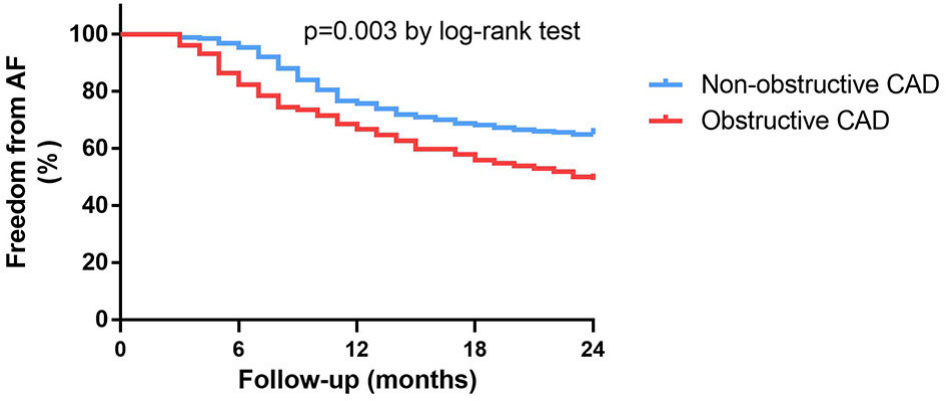
Kaplan-Meier survival curves for freedom from atrial fibrillation recurrence in patients with or without obstructive CAD at 24-month follow-up.

Of the 419 patients, approximately 61.1% (256 of 419) had no AF recurrence by the end of the 24-month follow-up period. After the ablation, angiotensin receptor blockers (ACEI/ARB) were used more frequently in patients with obstructive CAD than in patients without obstructive CAD (ACEI: 57.8 vs. 37.1%, *P* < 0.001; ARB: 93.1 vs. 54.3%, *P* < 0.001). The use of β-blockers was not significantly different between the two groups (Table 1). Patients without obstructive CAD showed a significantly higher proportion of freedom from AF than those in the obstructive CAD group (65 vs. 49%, *P* = 0.003; Figure 2). The adjusted regression analysis showed a lower rate of recurrence with an estimated hazard ratio of 1.72 [95% confidence interval (CI): 1.23–2.41, *P* = 0.001], favoring the group without obstructive CAD. The multivariate regression analysis showed that the predictors of AF recurrence after ablation were persistent AF (hazard ratio [HR]: 2.41; 95% CI: 1.72–3.38, *P* < 0.001) and left atrial diameter (HR: 2.59; 95% CI: 1.79–3.74; *P* < 0.001) (Table 2).

**Table 2:** Univariable and multivariable regression analyses of the recurrence of atrial fibrillation between the patients with obstructive CAD and without obstructive CAD.

**Variable**	**Univariable analysis**			**Multivariable analysis**		
	**Unadjusted HR**	**95%CI**	***P*-value**	**Adjusted HR**	**95%CI**	***P*-value**
Age (>60)	1.19	0.87–1.65	0.265			
Female, gender	1.13	0.82–1.57	0.451			
Dyslipidemia	1.29	0.95–1.77	0.105			
Renal insufficiency	0.98	0.24–3.96	0.983			
Diabetes mellitus	1.15	0.74–1.79	0.528			
Hypertension	1.35	0.99–1.85	0.054	1.00	0.69–1.45	0.984
CHA2DS2-VASC score (≥2)	1.35	0.99–1.84	0.056	1.16	0.80–1.66	0.424
Persistent AF	3.07	2.23–4.25	<0.001	2.41	1.72–3.38	<0.001
Obstructive CAD	1.66	1.19–2.31	0.003	1.72	1.23–2.41	0.001
Left atrial diameter (>42 mm)	3.44	2.42–4.90	<0.001	2.59	1.79–3.74	<0.001

*Values are reported as means ± SD or n (%). AF, atrial fibrillation; CAD, coronary artery disease; HR, hazard ratio; CI, confidence interval; SD, standard deviation*.

### Impact Factors for AF Recurrence in Patients With CAD

**Figure 3:**
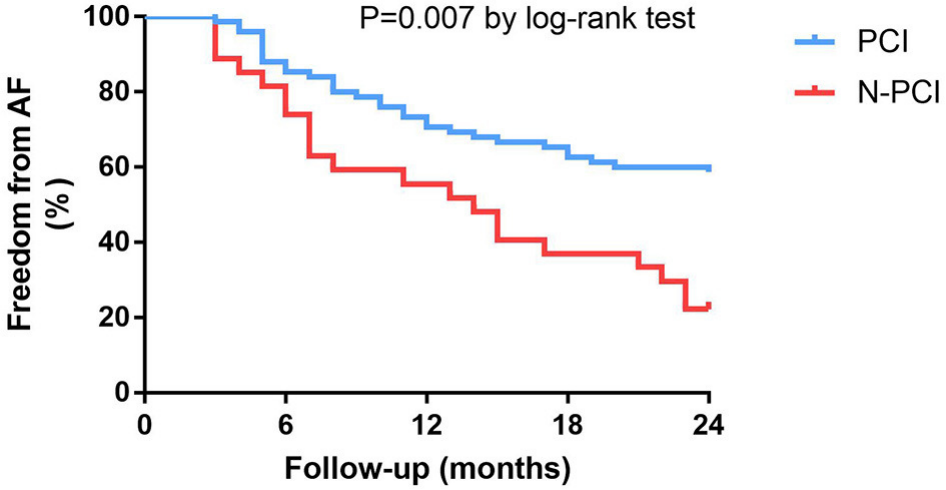
Kaplan-Meier survival curves for freedom from atrial fibrillation recurrence in patients with obstructive CAD at 24-month follow-up.

In general, 75 of 102 (73.5%) patients with obstructive CAD followed the doctor's recommendation and underwent successful PCI, while the other 27 (26.5%) patients chose to be treated with medications. Coronary angiography showed that 24 of 102 (23.5%) patients had more than one coronary artery stenosis. Patients with multi-vessel stenosis (≥2) had a higher rate of diabetes than patients with one coronary artery stenosis (66.6 vs. 8.9%, *P* < 0.001). Fourteen of the patients with multi-vessel stenosis received PCI treatment. The multivariable regression analysis showed that patients who chose PCI had a lower recurrence rate of AF than those without PCI (HR: 0.45; 95% CI: 0.25–0.80, *P* = 0.007, Figure 3). The other predictors of AF recurrence were multivessel stenosis (HR: 1.92; 95% CI: 1.04–3.54; *P* = 0.036) and left atrial diameter (HR: 2.56; 95% CI: 1.31–5.00; *P* = 0.006; Table 3).

**Table 3:** Univariable and multivariable regression analyses of the recurrence of atrial fibrillation in the patients with obstructive CAD.

**Variable**	**Univariable analysis**			**Multivariable analysis**		
	**Unadjusted HR**	**95%CI**	***P*-value**	**Adjusted HR**	**95%CI**	***P*-value**
Age (>60)	1.13	0.64–1.99	0.664			
Female, gender	0.71	0.39–1.28	0.262			
Dyslipidemia	1.32	0.73–2.78	0.357			
Renal insufficiency	0.70	0.09–4.99	0.713			
Hypertension	1.48	0.80–2.71	0.208			
CHA2DS2-VASC score (≥2)	1.38	0.79–2.42	0.255			
Diabetes mellitus	1.52	0.82–2.80	0.181			
Persistent AF	3.16	1.81–5.53	<0.001	1.83	0.99–3.38	0.053
PCI	0.40	0.23–0.71	0.002	0.450	0.25–0.80	0.007
Stenosis (≥2)	3.08	1.74–5.45	<0.001	1.92	1.04–3.54	0.036
Left atrial diameter (>42 mm)	3.10	1.65–5.83	<0.001	2.56	1.31–5.00	0.006

*Values are reported as means ± SD or n (%). AF, atrial fibrillation; PCI, percutaneous coronary intervention; HR, hazard ratio; CI, confidence interval; SD, standard deviation*.

### Subgroup Analysis of Recurrence in Patients With Non-obstructive CAD and Patients Without CAD

Of those who underwent coronary angiography, 140 (33.4%) were diagnosed as Non-obstructive CAD and 177 (42.2%) patients were diagnosed as without CAD. Non-obstructive CAD group had a similar recurrence of AF compared to Non-CAD group (40.7 vs. 30.5%, *P* = 0.075). The univariable proportional hazards analyses showed that there was no significant difference in AF recurrence between the Non-obstructive CAD group and Non-CAD group (HR: 1.33; 95% CI: 0.92–1.93; *P* = 0.128). The predictors of AF recurrence were persistent AF (HR: 2.41; 95% CI: 1.59–3.66; *P* < 0.001) and left atrial diameter (HR: 2.68; 95% CI: 1.72–4.17; *P* < 0.001) (Table 4).

**Table 4:** Univariable and multivariable regression analyses of the recurrence of atrial fibrillation in the patients without obstructive CAD.

**Variable**	**Univariable analysis**			**Multivariable analysis**		
	**Unadjusted HR**	**95%CI**	***P*-value**	**Adjusted HR**	**95%CI**	***P*-value**
Age (>60)	1.22	0.82–1.79	0.313			
Female, gender	1.33	0.90–1.96	0.152			
Dyslipidemia	1.03	0.68–1.57	0.873			
Renal insufficiency	0.97	0.13–7.01	0.983			
Diabetes mellitus	0.75	0.37–1.48	0.408			
Hypertension	1.20	0.82–1.74	0.333			
CHA2DS2-VASC score (≥2)	1.25	0.86–1.81	0.236			
Persistent AF	3.24	2.17–4.83	<0.001	2.41	1.59–3.66	<0.001
Non-obstructive CAD	1.38	0.95–2.00	0.08	1.33	0.92–1.93	0.128
Left atrial diameter (>42 mm)	3.53	2.30–5.39	<0.001	2.68	1.72–4.17	<0.001

*Values are reported as means ± SD or n (%). AF, atrial fibrillation; CAD, coronary artery disease; HR, hazard ratio; CI, confidence interval; SD, standard deviation*.

## Discussion

In the present study, we analyzed the relationship between coronary revascularization and AF recurrence in patients who underwent initial RFCA. The main findings are summarized as follows. First, we found that patients with obstructive CAD were less likely to achieve freedom from AF compared to patients without obstructive CAD. Second, for patients with CAD, coronary revascularization was associated with a lower recurrence rate after the initial RFCA. The multivariable regression analysis confirmed that coronary revascularization, multivessel stenosis, and left atrial diameter were important predictive factors for the recurrence of AF in patients with obstructive CAD after RFCA. Finally, there were no significant differences in AF recurrence between patients with Non-obstructive CAD (coronary stenosis <75%) and those without CAD.

### Coronary Artery Disease and Recurrence of AF

Since there are common risk factors in both diseases such as hypertension, diabetes mellitus, obesity, and sleep apnea, patients with AF often have coexisting CAD. The relationship between the AF substrate and chronic atrial ischemia has been demonstrated in previous animal experimental studies (17, 18). Right coronary artery occlusion promotes AF triggers and substrate formation, which facilitates the initiation and maintenance of AF (18). In addition, a substrate is formed to maintain AF, while acute atrial ischemia occurs after several hours (19). Acute atrial ischemia can lead to atrial effective refractory period shortening, increased conduction heterogeneity, and decreased conduction velocity (17, 20). Stable re-entrant sources at the border zone of an atrial myocardial infarction are associated with previous myocardial infarction fibrosis, which is an important reason for stabilizing re-entrant rotors. The myocardial fibers in the infarcted area give rise to conducting tracts that can set up a re-entry circuit. As described in an atrial myocardial infarction, fibrosis in the ventricle is also a factor in stabilizing re-entrant rotors (21), which can lead to fibrillatory conduction to maintain AF (18, 22). Another study from McLellan et al. (23) showed that ventricular fibrosis predicted the success of atrial fibrillation ablation.

The relationship between CAD and the outcome of AF after RFCA treatment has been analyzed in limited clinical studies. The Leipzig study, which enrolled 1,310 consecutive patients (12% patients with CAD), analyzed the impact of the presence and extent of CAD on recurrences after AF catheter ablation (24). The results demonstrated that there was no significant difference in arrhythmia recurrence rates in patients with and without CAD. In addition, the origin or extent of coronary stenosis was not associated with the recurrence ratio after catheter ablation in patients with or without CAD. Den Uijl's study recruited 125 AF patients and investigated the association of coronary atherosclerosis with the efficacy of RFCA during 12 month follow up (25). They found that the presence of coronary atherosclerosis is not associated with a higher risk for AF recurrence after RFCA. Another clinical study by Hiraya et al. revealed that there was a significantly higher rate of AF recurrence in patients with CAD than in patients without CAD (26). These results are inconsistent, highlighting the need for further research.

In the present study, persistent AF and left atrial diameter were predictors of AF recurrence after catheter ablation. We also found that patients with Non-obstructive CAD were significantly more likely to achieve freedom from AF than patients with obstructive CAD. The multivariate regression analysis showed that CAD was an independent risk factor for AF recurrence. CAD is a disease that could cause myocardial ischemia, which results in oxidative stress and atrial remodeling (27). Furthermore, myocardial ischemia can impair the left ventricle and increase the pressure in the left atrium, which causes changes in the tissue. These factors facilitate the initiation and recurrence of AF and reduce the success rate of RFCA. In the subgroup analysis, we compared AF recurrence after catheter ablation in patients with Non-obstructive CAD and those with normal coronary vessels. Interestingly, the results revealed that the recurrence of AF in patients with Non-obstructive CAD was similar to that of patients with normal coronary vessels, showing that patients with Non-obstructive CAD (vessel obstruction <75%) were not significantly different from those without coronary stenosis. It seems that the presence of coronary atherosclerosis does not impact the efficacy of RFCA because coronary atherosclerosis does not contribute to significant myocardial ischemia. Therefore, coronary atherosclerosis, which has no severe stenosis, has a limited impact on the recurrence of AF after RFCA, and other factors, such as persistent AF and left atrial diameter, limit the efficacy of RFCA.

### Coronary Revascularization and Recurrence of AF

Few studies have examined the impact of revascularization on AF recurrence in patients with CAD. Hiraya et al. investigated the role of revascularization in patients with CAD after initial PVI (26). The patients with CAD who had undergone PCI before PVI had a lower recurrence rate than those who did not undergo PCI. Our study demonstrated that coronary revascularization for CAD reduces the recurrence of AF after catheter ablation, and this finding concurs with that of Hiraya's study. Furthermore, we found that patients with multivessel stenosis (≥2) had a higher recurrence rate than patients with single-vessel stenosis. In addition to left atrial diameter and coronary revascularization, the multivariable regression analysis also showed that multi-vessel stenosis (≥2) was an independent predictive factor for AF recurrence in patients with CAD. Multi-vessel stenosis, which causes a higher degree of ischemia, is an important reason for the increased pressure in the left atrium, as discussed above. Coronary revascularization may decrease atrial ischemia and left atrial pressure in patients with CAD and AF. Therefore, revascularization for patients with CAD was associated with a lower recurrence rate of AF after RFCA.

### Limitations

This study had some inherent limitations. First of all, this study was a non-randomized study. There was a bias when selecting patients for coronary angiography. In many cases, it was difficult to distinguish chest symptoms between the discomfort caused by atrial fibrillation and angina due to CAD. Therefore, patients with atypical angina might not receive coronary angiography, On the other hand, it might cause a lower positive rate for patients undergoing coronary angiography. Secondly, when the coronary angiography results showed critical stenosis, although we used intravascular ultrasound to estimate the degree of obstruction, it still had the selection bias of CAD patients for revascularization. Thirdly, although we encouraged the patient to contact the physician in the case of suspected recurrence symptoms, data were limited by reliance on occasional and periodic ECG/Holter recording and clinical symptoms. Because asymptomatic arrhythmia episodes were difficult to be diagnosed, recurrence rates have been possibly underestimated.

## Conclusion

This study suggests that obstructive CAD is associated with a higher rate of AF recurrence. Additionally, for patients with CAD, coronary revascularization is related to a lower recurrence rate of AF after RFCA.

## Data Availability Statement

The original contributions presented in the study are included in the article/Supplementary Material, further inquiries can be directed to the corresponding author/s.

## Ethics Statement

The studies involving human participants were reviewed and approved by the Institutional Review Board (IRB) of First Affiliated Hospital of Zhengzhou University. The patients/participants provided their written informed consent to participate in this study.

## Author Contributions

The present study was performed by all authors. HT and XC contributed to the conception, design, and supervision of the study. XC, KZ, and JZ collected clinical information and performed data analysis. FQ and HL performed the clinical follow-up. XC wrote the manuscript. All authors discussed the results and contributed to the approval of the manuscript.

## Funding

This work was supported by the Joint Project of Medical Science and Technology Research of Henan (grant no. LHGJ20190099).

## Conflict of Interest

The authors declare that the research was conducted in the absence of any commercial or financial relationships that could be construed as a potential conflict of interest.

## Publisher's Note

All claims expressed in this article are solely those of the authors and do not necessarily represent those of their affiliated organizations, or those of the publisher, the editors and the reviewers. Any product that may be evaluated in this article, or claim that may be made by its manufacturer, is not guaranteed or endorsed by the publisher.
